# PROTOCOL: Protocol for corporate crime deterrence: An updated systematic review

**DOI:** 10.1002/cl2.1090

**Published:** 2020-05-17

**Authors:** Natalie Schell‐Busey, Melissa Rorie, Sally S. Simpson

**Affiliations:** ^1^ Department of Law & Justice Studies Rowan University Glassboro New Jersey; ^2^ Department of Criminal Justice University of Nevada—Las Vegas Las Vegas Nevada; ^3^ Department of Criminology and Criminal Justice University of Maryland College Park Maryland

## Abstract

This is the protocol for an updated Campbell review on corporate crime deterrence. Our overall objective is to identify and synthesize the extant empirical literature on *formal legal and administrative* prevention and control—that is, the actions and programs of government law enforcement agencies, legislative bodies, and regulatory agencies within a specific focus, as further discussed in this study. This review will consider all types of legal and regulatory practices as long as corporate crime prevention is part of the outcome. Other outcomes and information, if relevant, will also be collected.

## 1 BACKGROUND FOR THE REVIEW

Few subject areas span as many disciplinary boundaries as does corporate crime. Since Sutherland's famous Presidential Address to the American Sociological Association in 1939 and subsequent publication of *White Collar Crime* 10 years later, business scholars, economists, sociologists, political scientists, lawyers, and psychologists, and criminologists have speculated not just about the etiological origins of corporate crime but about the success of various strategies for its prevention and control. Yet, scholars and policy‐makers know very little about “what works, what doesn't, and what's promising” in this area. This is due to several related issues: (a) the ambiguity, scope, and complexity of the subject matter; (b) little systematic program or policy evaluation; (c) a lack of readily available and accessible data for research purposes which ultimately affects (d) the type and quality of research in this area.

Beginning with the first point, it is useful to define what *we* mean by corporate crime. Braithwaite (1984, p. 6) describes corporate crime as “the conduct of a corporation, or of employees acting on behalf of a corporation, which is proscribed and punishable by law.” Corporate crime, therefore, encompasses a wide array of illegal activities that are criminally, civilly, and administratively proscribed *and* which may be undertaken by individual managers/employees as well as by the firm (as an organizational actor). Corporate crimes generally are distinguished from other types of white‐collar offenses by the use of organizational resources and by who gains from the offense. Thus, when Raymond Scott Stevenson, head of Tyco's tax department, directed a series of transactions designed to reduce Tyco's state tax liability by back‐dating transactions to avoid reporting a $170 million dollar federal capital gain, he used organizational resources to “benefit” the company's bottom line.^1^

This distinction between white‐collar and corporate offending is by no means unambiguous. For instance, a top manager may utilize organizational resources to enrich him or herself—described as “collective embezzlement” by Calavita and Pontell (1991). In addition, although many acts of corporate crime are undertaken to achieve organizational goals, such acts may indirectly benefit the individual through promotion or salary bonuses. However, in accordance with Braithwaite's definition and consistent with our focus on corporate deterrence, we are interested in the kinds of behaviors typically characterized as “corporate” and not “white‐collar” offenses where the motivation for offending is organizational, not personal.

It is useful to categorize the kinds of offenses that meet our definitional criteria. Broadly conceived, corporate crimes in the United States^2^ can include the following categories of offenses: administrative noncompliance, environmental violations, financial violations, labor violations, manufacturing violations, and/or unfair trade practices (Clinard and Yeager, 1980, pp. 113–116). Similar to classifications of street crimes (e.g., violent crimes), each category contains a variety of specific offenses, often with distinct laws that define illegalities and provide remedies and sanctions for violators. For instance, unfair trade practices include monopolization, price‐fixing, unfair advertising, and price discrimination, among other illegal activities (Simpson, 1986). The Federal Trade Commission Act, Robinson‐Patman Act, and the Sherman‐Clayton Antitrust Act are some of the more significant pieces of legislation that define what constitutes unfair trade practices and the range of penalties for violators. Environmental violations are classified by different media (e.g., air, water, land) and statutes (e.g., Clean Water Act, Clean Air Act, Resource Conservation and Recovery Act). Similar to anti‐competitive illegality, some of these practices are defined as criminal offenses while others fall within the civil‐administrative realm. While many corporate offenses are financial, others are “violent” in nature, where human lives are lost and individuals injured (for instance, Occupational Health and Safety Administration violations).

A key feature of corporate offending is crime complexity. Although some offenses may be quite simple (bribery or accounting fraud), others often involve multiple interconnected actors and organizations, occur over long periods of time, and entail manipulating shell companies and billions of dollars (such as Enron). Corporate crimes also vary by seriousness. Egregious offenses can carry substantial criminal and civil sanctions while others are fairly minor “technical” violations (e.g., failure to submit a report to a regulatory agency). While definitional murkiness, breadth, and complexity make the phenomenon difficult to study, there are other barriers to research as well.

Perhaps the most salient barrier to research lies with the lack of high‐quality data. There is no UCR‐like national database that can be used to “measure” the corporate crime problem, nor are there any systematic procedures for identifying the “hidden” figure of crime. Most studies of corporate offending are qualitative, case study investigations of sensational events. There are only a handful of systematic scientific studies of corporate offending (including Sutherland's original study) because most federal agencies that fund criminological research (e.g., National Institute of Justice) focus on “street” crime. These agencies are also more apt to fund evaluation research on programs and policies in these same areas. We therefore have learned a great deal about the successes or failures of drug courts, boot camps, or gun seizures, but relatively little about whether internal compliance systems (such as ethics training, randomized audits, hotlines) reduce illegal behavior by companies or if criminal prosecution promotes corporate deterrence and compliance better than civil litigation or regulatory interventions.

Because the subject matter crosses so many disciplinary boundaries, there are studies and evaluations outside of criminology and criminal justice that inform a systematic review in this area. Thus, our search encompasses other disciplines, such as psychology and business. In our initial review, we narrowed the scope of studies to those that examine the effectiveness of legal restraints (including laws, official sanctions, and regulatory actions). Therefore, this update is restricted to that particular domain as well.

## 2 OBJECTIVES OF THE REVIEW

Our overall objective is to identify and synthesize the extant empirical literature on *formal legal and administrative* prevention and control—that is, the actions and programs of government law enforcement agencies, legislative bodies, and regulatory agencies within a specific focus, as discussed below. This review will consider all types of legal and regulatory practices as long as corporate crime prevention is part of the outcome. Other outcomes and information, if relevant, will also be collected.

Second, we need to assess the “quality” of this evidence (i.e., the kinds of studies and data that exist to answer our research questions) to determine whether a meta‐analytic review is possible in this domain.

Once we have retrieved and fully coded relevant publications (including the calculation of effect sizes), we plan to focus on the effectiveness of the identified strategies and programs. Specifically, we will address the following questions:

- Which kinds of interventions (prevention and control) lower the risk of corporate offending?
- Do different types of interventions have different kinds of effects?
- Do effects vary by unit of analysis (e.g., manager vs. firm?)
- Do effects vary by population characteristics (e.g., big firms vs. small firms, public vs. private, profit‐seeking vs. non‐governmental organizations, etc.)
- Do interventions have different effects by offense type?
- How are studies conceptualizing deterrence (i.e., general vs. specific, objective vs. perceptual)?
- How are studies measuring deterrence (e.g., self‐reported perceptions, arrest rates, etc.) and do these different measures produce different effects?
- At what point in the legal process is deterrence being assessed (e.g, during investigation, sentencing, etc.)?

## 3 METHODS

### 3.1 Criteria for inclusion and exclusion of studies in the review

We will conduct a search for articles using a set of search terms aimed specifically at legal and regulatory policies and sanctions (see Section 3.2) and focus on studies that involve both corporate crime behaviors and are empirical (including studies using either quantitative and qualitative methods) in nature.

After retrieving those articles, we will further cull the articles to find those we consider to be eligible for coding. Eligible articles are those that meet the following criteria:

(1) The study was an evaluation of a corporate crime prevention/control strategy in the legal or administrative domains (i.e., deterrence resulting from effective regulations, fines, regulatory inspections, etc.).
(2) The study includes a comparison group (or a preintervention comparison period in the case of pre–post studies) that did not receive the treatment condition. Studies may be experimental, quasi‐experimental, or pre–post evaluations. If the study does not include a treatment *group*, does it report standardized regression coefficients/Pearson correlations if the treatment is measured continuously?
(3) The study reports on at least one crime/misconduct outcome. In accordance with our broad definition of corporate crime (see Section 1), the outcome of interest may be one of a wide range of criminal behaviors, regulatory violations, or civil violations.
(4) The study is written in English, but may be cross‐national.
(5) The study was published after 2011 and before 2019. Plans to update the study after this current review are described in Section 5.
(6) Published and unpublished studies are included.

### 3.2 Search strategy for identification of relevant studies

Our search will include published and unpublished articles, reports, documents, and other readily available sources. The studies will be identified via an exhaustive search of multiple online databases and other sources using 57 search terms. These databases and legal/deterrence search terms are described below. In addition to the online searches, we will review the bibliographies of seminal articles/books that address corporate crime deterrence, prevention, and control. We also plan to email the final list of articles deemed eligible for coding to leading corporate crime scholars in case we have missed other important sources.

The databases used in our search for *published* articles include:

- Social Work Abstracts
- ABI
- PsycINFO
- Sociological Abstracts
- ERIC
- Criminal Justice Abstracts
- Worldwide Political‐Science Abstracts
- Business Source Elite
- EconLit
- PAIS International
- WorldCat FirstSearch

After conducting the search for published documents described above (including reviewing the articles’ bibliographies and later articles citing eligible studies in Web of Science), we will conduct subsequent searches for unpublished and missed published documents in the following sites^3^:

- Google Scholar
- Digital Dissertation Database
- Financial Crimes Enforcement Network Website
- Ministry of Finance Netherlands Website
- Australia Institute of Criminology
- DOJ
- SEC
- EPA
- FTC
- FinCen
- UNODC
- World Bank

The search terms used to collect studies from the above databases are given below:

1. Sanction AND Fraud
2. Sanction AND “Anti‐competitive Behavior”
3. Sanction AND Antitrust
4. Sanction AND Corruption
5. Sanction AND “Business Crime”
6. Sanction AND “Business Misconduct”
7. Sanction AND “Business Violations”
8. Sanction AND “Corporate Manslaughter”
9. Sanction AND “Corporate Crime”
10. Sanction AND “Corporate Misconduct”
11. Sanction AND “Corporate Violations”
12. Sanction AND “Environmental Crime”
13. Sanction AND “Organizational Crime”
14. Sanction AND “Organizational Misconduct”
15. Sanction AND “Organizational Violations”
16. Sanction AND “Ethical Business Culture”
17. Sanction AND “Unethical Conduct”
18. Sanction AND “Unethical Behavior”
19. Sanction AND “White Collar Crime”
20. Fine AND Fraud
21. Fine AND “Anti‐competitive Behavior”
22. Fine AND Antitrust
23. Fine AND Corruption
24. Fine AND “Business Crime”
25. Fine AND “Business Misconduct”
26. Fine AND “Business Violations”
27. Fine AND “Corporate Manslaughter”
28. Fine AND “Corporate Crime”
29. Fine AND “Corporate Misconduct”
30. Fine AND “Corporate Violations”
31. Fine AND “Environmental Crime”
32. Fine AND “Organizational Crime”
33. Fine AND “Organizational Misconduct”
34. Fine AND “Organizational Violations”
35. Fine AND “Ethical Business Culture”
36. Fine AND “Unethical Conduct”
37. Fine AND “Unethical Behavior”
38. Fine AND “White Collar Crime”
39. “Regulatory Policy” AND Fraud
40. “Regulatory Policy” AND “Anti‐competitive Behavior”
41. “Regulatory Policy” AND Antitrust
42. “Regulatory Policy” AND Corruption
43. “Regulatory Policy” AND “Business Crime”
44. “Regulatory Policy” AND “Business Misconduct”
45. “Regulatory Policy” AND “Business Violations”
46. “Regulatory Policy” AND “Corporate Manslaughter”
47. “Regulatory Policy” AND “Corporate Crime”
48. “Regulatory Policy” AND “Corporate Misconduct”
49. “Regulatory Policy” AND “Corporate Violations”
50. “Regulatory Policy” AND “Environmental Crime”
51. “Regulatory Policy” AND “Organizational Crime”
52. “Regulatory Policy” AND “Organizational Misconduct”
53. “Regulatory Policy” AND “Organizational Violations”
54. “Regulatory Policy” AND “Ethical Business Culture”
55. “Regulatory Policy” AND “Unethical Conduct”
56. “Regulatory Policy” AND “Unethical Behavior”
57. “Regulatory Policy” AND “White Collar Crime”

The first task involving these searches is to keep track of the number of “hits” each search term reveals within each database. Next, we will review all titles and abstracts to determine: (a) whether the article is relevant to our study; and (b) whether the article is quantitative or not. After that we will sort the empirical articles by keywords across search engines to eliminate article redundancy between search engines. We will then identify articles that are eligible for complete coding based on the criteria defined in Section 3.1.

### 3.3 Description of methods used in the component studies

We include studies that use a wide variety of methods, but will concentrate on identifying studies in which a treatment group that was subject to a specific legal restriction was compared to a control group that was not. Studies can be experimental, quasi‐experimental, or pre–post evaluations. We will also include observational studies in which groups were constructed by natural means (e.g., analyzing adjacent jurisdictions). In the case of observational data, we will include studies that report standardized regression coefficients or Pearson's correlations as well as those that have enough information to allow the calculation of an effect size.

The studies included will use various samples consisting of individuals (e.g., employees, students, CEOs), corporations, or geographical areas. These different units of analysis will be kept separate for the purpose of our analyses.

Given our definition of corporate crime,^4^ the outcome variables included in our study will be very broad. Some examples of the outcomes (but not an exhaustive list) include variations in pollution emissions, official records of compliance with regulations (e.g., environmental, employment, OSHA), recidivism, safety violations/compliance, number of financial transactions, perceived intentions to offend, perceptions of ethicality of behaviors, injuries from safety violations or environmental accidents, convictions, citations, noncompliant inspections, compliance measures (e.g., self‐ratings), accuracy of regulatory records, complaints (e.g., about consumer fraud), and perceptions of enforcement effectiveness.

### 3.4 Criteria to ensure we are only using independent findings

Many studies report more than one outcome that is relevant to our domain of interest and many authors publish more than one article using data from the same sample. In order to statistically analyze our coded articles properly, we must make sure that the effect sizes we calculate come from independent samples. To ensure that this is the case, we will enter the articles into a data file (using Microsoft Excel). As the coders code the articles, they will note where a sample may have overlapped with another study. For each study, we will differentiate truly unique outcomes derived from the same sample, and then will combine multiple effect sizes describing the same outcome from the same sample. Before completing our analyses, we will review all of the sample characteristics from the population of studies to verify that any effect sizes from different studies utilizing the same sample are combined for our final analysis.

### 3.5 Details of study coding categories

The coding protocol (Supporting Information Appendix A) has been updated to reflect the narrower focus that was used for the original meta‐analysis and that will be used for this update as well. In the coding protocol, the variable named “TREATMENT” (p. 18 of the current document) provides all potential descriptions of the treatment program; those treatments falling under (a) Law, (b) Official Sanction/Fine, (c) Regulatory Policy, or (d) Non‐punitive action by regulatory agency (e.g., warning letter, cease and desist order).

The protocol includes codes used to describe the source of the study (Section I of Appendix A; e.g., country of publication, journal's disciplinary area), characteristics of the study (Section II; e.g., randomized experiment or not, start/end date of data collection, concerns about validity), sample characteristics (Section III; e.g., whether individuals or corporations), the methods and procedures used by the study authors (Section IV; e.g., use of a control group), descriptions of the independent variable (Section V; e.g., construct and operationalization), descriptions of the dependent variable (Section VI; e.g., construct and operationalization), effect size data (Section VII; i.e., coding the data provided that will be employed to calculate an effect size), and then conclusions made by the study authors (Section VIII). There are also shaded boxes at the very end that describe the various types of effect sizes and relevant statistics needed for future analysis.

Articles will be coded by two coders, who will input all data into a Microsoft Excel spreadsheet. An initial coding session will be completed in which 20 articles are coded in order to calculate interrater reliabilities. Coders will then resolve differences between the two databases. If a decision rule is needed, it will be added to those already at the end of the coding protocol. The coders will then review another 20 articles following the new decision rules until an acceptable interrater reliability is established for most variables (those not reaching either a *κ* value or Pearson's correlation value of 0.70 will not be used in further analyses). The coders will then split the rest of the articles for independent coding. No changes will be made to the coding sheet after an acceptable interrater reliability is established.

### 3.6 Statistical procedures and conventions

Due to the breadth of the outcomes included in our systematic review, we will likely be coding various forms of data that will result in multiple types of effect sizes being calculated. For example, dichotomous outcomes will likely be calculated as an odds ratio, while continuous outcomes in a two‐group comparison will likely result in a standardized mean‐difference effect size. However, we also will have data in which both the independent variable and dependent variable are continuous; such data is used to calculate a product‐moment correlation effect‐size statistic. When reporting the results, we will only compare similar effect sizes to each other and combine within types for the appropriate analysis.

Following Lipsey and Wilson (2001), mean effect sizes and the homogeneity of effects across studies will be computed using the inverse variance weight method. We assume a random‐effects model and will calculate variance components accordingly. Computations will be run using Stata macros provided by D. B. Wilson. Sample output from these macros from a previous analysis (Rorie, Schell‐Busey, & Simpson, 2009) is presented in Supporting Information Appendix B.

### 3.7 Treatment of qualitative research

Although we consider all empirical studies (using either qualitative or quantitative methods) in this review, we only use studies that allow us to code usable quantitative data. Therefore, we do not currently plan on including purely qualitative studies in our systematic review.^5^

## 4 TIMEFRAME

We have already begun collecting the new (since 2011) published studies, but we still need to complete that search and then begin searching for new unpublished studies. We hope to have a complete bibliography of published and unpublished studies (through 2018) by December 2019.

After the bibliography is complete, we will begin checking the articles for eligibility, which we hope to complete by the end of February 2020. Once we have a list of eligible articles, we will begin coding the data for the eligible studies and then calculate effect sizes where possible. This entails the following steps:

1. Identify where we have enough information to calculate an effect size. Where we don't have enough information, we will attempt to email the authors and collect that information.
2. Using the Lipsey/Wilson decision tree, determine what the appropriate effect size calculation will be—whether it will be OR, *d*, or *r*.
3. Calculate unbiased effect sizes and standard errors in Excel using formulas in the Lipsey/Wilson book.
4. As needed (for specific projects), plug individual effect sizes into Stata and use macros to calculate overall effect sizes.

The projected completion date for calculating effect sizes is August 2020, after which we will begin work on the analysis and the written products, including a Campbell Collaboration report. We hope to have a written report to the Campbell Collaboration by August 2021.

## 5 PLANS FOR UPDATING THE REVIEW

Once we submit the written report to the Campbell Collaboration and at least one journal publication, we will begin work on updating the review. We plan on updating the review every 3 years in accordance with Campbell Collaboration guidelines.

## Supporting information

Supporting information

## Footnotes

^1^

http://www.usdoj.gov/usao/fls/PressReleases/060921‐04.html

^2^
These categories of offenses are particular to the United States. Other countries, especially in the west, may have similar classifications, but the laws and punishments are not necessarily comparable.

^3^
For the update, we are reducing the number of websites used for the unpublished search to focus only on those that produced a significant number of hits for the first meta‐analysis. We may further narrow the websites used to the first five listed if the others prove inefficient for the purpose of locating empirical studies on corporate crime interventions during our years of interest. The first five websites allow searches using string terms and filtered searches to specify our dates of interest while the other sites do not allow such specific searches.

^4^
For our purposes, we will include studies that examine criminal and regulatory violations by corporations or their employees. The majority of corporate offenses are handled be regulatory agencies, like the EPA and OSHA. Thus, a focus on strictly criminal behaviors would limit this study and miss a great deal of corporate misconduct. According to Braithwaite (1984), corporate crime is the “conduct of a corporation, or of employees acting on behalf of a corporation, which is proscribed and punishable by law” (p. 6). This offense‐based definition encompasses a wide range of behaviors such as antitrust offenses, intentionally polluting the environment, unsafe labor practices, and tax and securities violations. It also includes individual‐level behaviors as well as corporate actions.

^5^
In our review of the empirical literature thus far, we have found no qualitative studies that produce data that is usable for meta‐analytic purposes.

## References

[cl21090-bib-0001] Braithwaite, J. (1984). Corporate crime in the pharmaceutical industry. London: Routledge and Kegan Paul.

[cl21090-bib-0002] Calavita, K. , & Pontell, H. N. (1991). “Other's people's money” revisited: Collective embezzlement in the savings and loan and insurance industries. Social Problems, 38(1), 94–112.

[cl21090-bib-0003] Clinard, M. B. , & Yeager, P. C. (1980). Corporate crime. New Brunswick, NJ: Transaction Publishers.

[cl21090-bib-0004] Lipsey, M. W. , & Wilson, D. B. (2001). Practical meta‐analysis. Thousand Oaks, CA: SAGE Publications.

[cl21090-bib-0005] Rorie, M. , Schell‐Busey, N. , & Simpson, S. S. (2009). *All bark and no bite?: Comparing the effectiveness of internal compliance, external controls, and legal authorities as corporate watchdogs*. Presented at the American Society of Criminology Annual Conference, Philadelphia, PA.

[cl21090-bib-0006] Simpson, S. S. (1986). The decomposition of antitrust: Testing a multi‐level, longitudinal model of profit‐squeeze. American Sociological Review, 51(6), 859–875.

