# Supplementary material for: PROTOCOL: Protocol for corporate crime deterrence: An updated systematic review
Source: Campbell Syst Rev. 2020 May 17;16(2):e1090. doi: 10.1002/cl2.1090 (PMC8356332; doi:10.1002/cl2.1090)
Supplement: Supplementary file 1 — Supporting information [file CL2-16-e1090-s001.docx]

**Appendix A: Corporate Crime Systematic Review Coding Sheet**

**CC Meta Analysis Coding Sheets: Study-Level Coding Protocol**

**Bibliographic Reference (APA format):__________________________________________________________________________________________________________________________________________________________________________________________________________________________________**

| **I. Source Descriptors** | | | | | | |  |  |
| --- | --- | --- | --- | --- | --- | --- | --- | --- |
| **Variable Name** | **Code** | | | **Item** | | |  |  |
| ID |  | | | 1. Study ID number:    - First 3 letters of first author’s last name followed by year    - If duplicates, add an “A” or “B” based on alphabetical order of titles | | |  |  |
| PUBTYPE |  | | | 2) Type of Publication:   \| 1. Book \| 2. Book chapter \| \| --- \| --- \| \| 3. Journal article \| 4. Thesis or dissertation \| \| 5. Government report (state/local) \| 6. Government report (federal) \| \| 7. Working paper \| 8. Conference paper \| \| 9. Regulatory Agency report \| 10. Corporate Report \| \| 11. Other (specify) \|  \| | | |  |  |
| PUBTYPE_OTH |  | | | 2b) Type of Publication—specify other publication type: | | |  |  |
| YEAR |  | | | 3) Year of Publication | | |  |  |
| DISCIPLINE |  | | | 4) Disciplinary Affiliation of Publication/Journal^[[1]](#endnote-1)^:  1. Criminology  2. Sociology  3. Business/Marketing  4. Political Science  5. Environmental Science/Biology  6. Psychology  7. Public Policy  8. Economics  9. Other  10. Multiple disciplines (list under DISC_OTH) | | |  |  |
| DISC_OTH |  | | | 4b) Disciplinary Affiliation of Publication/Journal—specify other discipline: | | |  |  |
| FUNDING |  | | | 5) Source of funding for the research:  0. No funding/None reported  1. Government agency  2. University  3. NGO/Non-profit  4. Private business  5. Other (specify) | | |  |  |
| FUND_OTH |  | | | 5b) Source of funding for the research—specify other: | | |  |  |
| NAT_PUB |  | | | 6) Country of Publication | | |  |  |
| DATE |  | | | 7) Date coded | | |  |  |
| CODER ID |  | | | 8) Coder:  1. Grad Assistant A (Rorie)  2. Grad Assistant B (Schell-Busey) | | |  |  |
| **II. Study Characteristics** | | | | | | |  |  |
| STUDYTYPE |  | | | 1. Type of study:   1. Randomized experiment (in-basket or lab; e.g., conditions are randomized at the individual level or everyone receives the same survey)  2. Randomized experiment (vignette survey; e.g., conditions within scenarios are randomized)  3. Nonequivalent control group (quasi-experimental)—has a comparison group that is not randomly assigned (e.g., matched pairs comparison or propensity score matching)  4. Time-series/pre-post test (no control group)  5. Time-series/pre-post test (with control group)  6. Non-experimental (i.e., multiple regression or correlation)  7. Other (specify) | | |  |  |
| STTYPE_OTH |  | | | 1b) Type of study—specify Other: | | |  |  |
| STARTDATE |  | | | 2) Date Range of Research: First year of data | | |  |  |
| ENDDATE |  | | | 3) Date Range of Research: Last year of data | | |  |  |
| NAT_STUD |  | | | 4) Country where study conducted: | | |  |  |
| NUMOUT |  | | | 5) Number of crime/misconduct outcomes reported in study^[[2]](#endnote-2)^ | | |  |  |
| UOA |  | | | 6) What is the unit of analysis in this study (i.e., the type of outcome)?   1. Individual decision-making/behavior 2. Company decision-making/behavior 3. Geographic area (e.g., state, country) 4. Other (specify) | | |  |  |
| UOA_OTH |  | | | 6b) What is the unit of analysis in this study? Specify other: | | |  |  |
| DATARLBTY |  | | | 7) Did the researcher empirically assess the reliability of the data collected?  1. Yes  0. No | | |  |  |
| DATAVLDY |  | | | 8) Did the researcher assess the validity of the data collected (e.g., discussed whether measures used accurately represented the construct of interest)?  1. Yes  0. No | | |  |  |
| DATAPROB |  | | | 9) If the researcher noted any concerns about the data, please describe here: | | |  |  |
| DETTYPEConc |  | | | 10) How was deterrence conceptualized:   1. General deterrence 2. Specific deterrence 3. Other (e.g., perceptual, objective, focused) 4. Multiple types of deterrence mentioned   888. No specific form of deterrence mentioned | | |  |  |
| DETTYPEConc_OTH |  | | | 11) If DETTYPE = 3 or 4, please explain what type(s) of deterrence the manuscript examined. | | |  |  |
| DETTYPEStage |  | | | 10) At what stage was deterrence being assessed?:   1. Pre-arrest (e.g., hypothetical scenarios) 2. Arrest 3. Prosecutorial Decision (e.g., NPA/DPA versus full prosecution) 4. Sentencing (e.g., effect of fine, incarceration)   888. No specific form of deterrence mentioned | | |  |  |
| DETTYPEStage_OTH |  | | | 11) If DETTYPE = 3 or 4, please explain what type(s) of deterrence the manuscript examined. | | |  |  |
| **III. Sample Descriptors** | | | | | | |  |  |
| SAMPLEN |  | | | 1) Sample size | | |  |  |
| SAMP_INDCOR |  | | | 2) Does the sample consist of individuals or corporations?  1. Individuals  2. Corporations  3. Other (specify; e.g., court cases) | | |  |  |
| SAMP_OTHER |  | | | 2b) Does the sample consist of individuals or corporations? Specify other: | | |  |  |
| SAMP_MIX |  | | | 3) Was the sample drawn from more than one organization?  1. Yes  0. No | | |  |  |
| **If the sample consists of individuals, answer the following questions. Otherwise, skip to question #9:** | | | | | | |  |  |
| AGE |  | | | 4) Mean Age of Sample (if mean age cannot be determined, enter 888) | | |  |  |
| RACE |  | | | 5) Predominant Race of sample  1. Mostly white  2. Mostly black  3. Mostly hispanic  4. Mostly asian  5. Mixed, none more than 50%  6. Mixed, cannot estimate proportion  888. Unknown/Not reported | | |  |  |
| SEX |  | | | 6) Predominant Sex of sample  1. 60% or more male  2. 60% or more female  3. Even mix of male and female  888. Unknown/Not reported | | |  |  |
| MGMT |  | | | 7) Predominant management level of sample:  1. 60% or more non-managerial employee  2. 60% or more middle managers or supervisors  3. 60% or more CEO/Executives (or highest-level employees such as law firm partners)  4. Even mix of multiple levels  5. Other (Specify)  888. Unknown/Not reported | | |  |  |
| MGMT2 |  | | | 7b) Management level of sample—specify other: | | |  |  |
| PRTCPNT |  | | | 8) Who were the participants of the study?   1. Unemployed students 2. Working students 3. Both unemployed and working students 4. Professionals 5. Both students and professionals | | |  |  |
| EDUCATION |  | | | 9) Predominant education level of sample  1. 60% or more: High school degree or less  2. 60% or more : Some college education (or currently in college)  3. 60% or more: College graduates  4. 60% or more: Some graduate education (or currently in graduate program)  5. 60% or more: Completed graduate degree  6: Even mix of multiple education levels  888. Unknown/Not reported | | |  |  |
| EMPLENGTH |  | | | 10) Length of employment of the target population:________  1. No work experience  2. Less than 5 years  3. Between 5 – 10 years  5. More than 10 years  6. Multiple levels of experience included in sample  888. Unknown/Not reported | | |  |  |
| INDUSTRY |  | | | 11) From what industry was the sample drawn? (choose all that apply)   \| 1. Agriculture \| 2. Accounting \| \| --- \| --- \| \| 3. Advertising \| 4. Airline \| \| 5. Banking \| 6. Biotechnology \| \| 7. Computer/Technology \| 8. Consumer products \| \| 9. Defense \| 10. Education \| \| 11. Energy \| 12. Food, beverage, or tobacco \| \| 13. Health care \| 14. Investment banking \| \| 15. Legal \| 16. Manufacturing \| \| 17. Marketing/Business \| 18. Pharmaceuticals \| \| 19. Real Estate \| 20. Retail \| \| 21. Securities and Commodities \| 22. Service \| \| 23. Telecommunications \| 24. Transportation \| \| 888. Unknown/Not reported \| 25. Other (specify) \| \| 26. Multiple categories (list under IND_OTH) \| \| | | |  |  |
| IND_OTH |  | | | 11b) From what industry was the sample drawn? Specify other: | | |  |  |
| **If the sample consists of corporations, please answer the following questions:** | | | | | | |  |  |
| COMPSIZE |  | | | 12) Average number of employees in sample companies (if UOA is firm and information is not given, record 888) | | |  |  |
| COMPPROF |  | | | 13) Average profit of companies in sample (not given = 888) | | |  |  |
| COMPSALES |  | | | 14) Average annual sales of companies in sample (not given = 888) | | |  |  |
| **IV. Methods and Procedures** | | | | | | |  |  |
| RANDOM |  | | | 1. Was the sample randomly selected?    1. Yes 2. No   888. Unclear or not reported | | |  |  |
| SAMPPROC |  | | | 1. Sampling procedures    1. Random probability sample    2. Stratified random sample    3. Matched-pairs    4. Snowball sampling    5. Convenience sample (drawn from individuals to which researchers have easy access)    6. Secondary data analysis (without specification of sampling procedures)    7. Other (specify) | | |  |  |
| SAMPPR_OTH |  | | | 2b) Sampling procedures—specify other: | | |  |  |
| SURVEY |  | | | 1. Survey design    1. Mail    2. Phone    3. Face-to-face Interview    4. Other (specify)   777. Not applicable (not a survey) | | |  |  |
| SURVEY_OTH |  | | | 3b) Survey design—specify other: | | |  |  |
| CROSSSEC |  | | | 1. Is the research design cross-sectional or longitudinal?    1. Cross-sectional (including pooled cross-sections or lagged independent variables)    2. Longitudinal (i.e., the dependent variable is measured at more than one point in time to assess change over time) | | |  |  |
| BIAS |  | | | 1. Did the authors assess the differences between survey respondents’ and non-respondents’ background characteristics?    1. Yes 2. No   777. Not applicable (not a survey) | | |  |  |
| BIAS_YES |  | | | 5b) If yes, were significant differences found between responders’ and nonresponders’ background characteristics?   1. Yes 2. No   777. Not applicable | | |  |  |
| BIAS_ADD |  | | | 5c) If yes, what did the authors do to address these differences? | | |  |  |
| RESPRATE |  | | | 6) Response rate to survey (777 if not a survey) | | |  |  |
| ATTRITION |  | | | 7) If longitudinal, rate of attrition (put 777 if not a longitudinal panel survey) | | |  |  |
| SIGLEVEL |  | | | 8) Level of statistical significance used (usually .05) | | |  |  |
| CONTROL |  | | | 9) Nature of control group  1. Randomly assigned—no treatment  2. Randomly assigned—alternative treatment  3. Natural experiment—no treatment  4. Natural experiment—alternative treatment  5. Time-series—pre/post  6. Propensity-score matching/Matched pairs  7. No control group (not an experimental design or not a listed QED) | | |  |  |
| PRETEST |  | | | 10) Did the authors assess pre-test differences between tx/control groups?   1. Yes 2. No | | |  |  |
| PRTST_DIFF |  | | | 10b) If so, were differences found between groups?   1. Yes 2. No | | |  |  |
| PRTST_ADD |  | | | 10c) If yes, what did the researchers do to address these differences? | | |  |  |
| **V. Description of Independent Variable** | | | | | | | | |
| TREATMENT | | |  | | | 1) What form did the treatment take?^[[3]](#endnote-3)^  1. Law (including case law)  2. Official sanction/Fine (e.g., conviction, prosecution, prison) OR punishment avoidance (e.g., acquittal)  3. Regulatory Policy (e.g., inspections, agency resources, deregulation)  4. Non-punitive action by regulatory agency (e.g., warning letter, cease and desist order)  5. Other (specify)  6. Multiple treatments involved | | |
| TREAT_DES | | |  | | | 1b) Brief description of treatment: | | |
| TREAT_BIN | | |  | | | 1c) Was the independent variable binary or continuous?   1. Binary 2. Continuous | | |
| TREATCON_DES | | |  | | | 1. Description of continuous independent variable measurement: | | |
| IV_SOURCE | | |  | | | 4) What data sources were used to measure the independent variables? (Select all that apply)   1. Official data 2. Self-report data (e.g., surveys or interviews) 3. Observations/site visits of places or environments 4. Other (specify) | | |
| IVSRCE_OTH | | |  | | | 4b) What data sources were used to measure the independent variables? Specify other: | | |
| CONTROLS | | |  | | | 5) Did the authors control for potentially spurious variables?  1. Yes  0. No | | |
| **VI. Dependent Variable Descriptors** | | | | | | | |  |
| OUTCM_ACT | |  | | | 1) Did the outcome describe *actual* behavior (e.g., arrests) or *intentions* (e.g., hypothetical situations)?   1. Actual behavior 2. Intentions/Opinions about behavior/Lab setting 3. Both | | |  |
| OUTCM_DSC | |  | | | 1b) Brief description of outcome^[[4]](#endnote-4)^: | | |  |
| OUTCMDTA | |  | | | 2) What type of data was used to measure the outcome covered on this coding sheet?   1. Official data 2. Self-report data (e.g., surveys or interviews) 3. Observations/site visits of places or environments 4. Other (specify) | | |  |
| OUTDTA_OTH | |  | | | 2b) What type of data was used to measure the outcome covered on this coding sheet? Specify other: | | |  |
| OUTMSRE | |  | | | 3)How was the DV measured?  1. Scale—1 item  2. Composite  3. Raw number of violations (or rates: #/unit of opportunity)  4. Dichotomous measure  5. Other (specify)—e.g., dollar amounts | | |  |
| OUTMSRE_OTH | |  | | | 3b) How was the DV measured? Specify other: | | |  |
| OUTMSRE_DES | |  | | | 4)Description of continuous outcome measure: | | |  |
| ILL_UNETH | |  | | | 5) Is the DV measured using illegal or unethical behavior?^[[5]](#endnote-5)^  1. Illegal (e.g., can be sanctioned by law enforcement or regulatory sanctions, or is subject to auditing)  2. Unethical (morally ambiguous but not subject to sanctions)  3. Both  4. Other (specify): (Unclear whether sanctionable/only related to company policies)  888. Unknown/Not reported | | |  |
| ILLUNETH_OTH | |  | | | 5b) Is the DV measured using illegal or unethical behavior? Specify other: | | |  |
| COMP_SOC | |  | | | 1. Is the victim of the offense the company or general society? 2. Company 3. Society 4. Other (specify) | | |  |
| COMP_SOC_OTH | |  | | | 6b)Does the behavior affect the company or society, according to Akers’ (1977) list? Specify other: | | |  |
| **II. Effect Size Data^[[6]](#endnote-6)^** | | | | | | | |  |
| ATT_PROB | |  | | | 1) Was attrition a problem for this outcome?  1. Yes  0. No  777. Not Applicable (not a panel survey)  888. Not reported/unknown | | |  |
| ATT_CASES | |  | | | 2) If attrition was a problem, how many cases were lost? | | |  |
| ATT_REAS | |  | | | 3) If attrition was a problem, why were cases lost? | | |  |
| RAWDIFF | |  | | | 4) Raw difference (i.e., means or frequencies) favors (i.e. shows more success for):  1. Treatment group (or post period)  2. Control group (or pre period)  3. Neither (exactly equal)  888. Unknown  777. Not applicable | | |  |
| SIGDIFF | |  | | | 5) Did a test of statistical significance indicate statistically significant differences between either the control and treatment groups or the pre and post tested treatment group?  1. Yes  0. No  888. Unknown  777. Not applicable | | |  |
| STANDES | |  | | | 6) Was a standardized effect size reported?  1. Yes  0. No | | |  |
| ES | |  | | | 7) If yes, what was the effect size | | |  |
| ES_PAGE | |  | | | 8) If yes, page number where effect size data is found | | |  |
| NOES | |  | | | 9) If no, is there data available to calculate an effect size?  1. Yes  0. No | | |  |
| NOES_DATA | |  | | | 10) Type of data effect size can be calculated from:^[[7]](#endnote-7)^  1. Means and standard deviations  2. *t*-value or *z*-value  3. *F*-value  4. Chi-square (df=1)  5. Frequencies or proportions (dichotomous)  6. Frequencies or proportions (polychotomous)  7. Pre and post  8. Standardized regression coefficients  9. Unstandardized regression coefficients  10. Correlations (Pearson’s *r*)  11. Other (specify) | | |  |
| NOES_OTH | |  | | | 10b) Type of data effect size can be calculated from—specify other: | | |  |
| NOES_REG | |  | | | 10c) If the data presented is an *unstandardized* regression coefficient, what type of regression was used?   1. OLS 2. Logistic 3. Tobit 4. Poisson 5. Other (specify) 6. Ordered logit | | |  |
| NOES_REG2 | |  | | | 10d) If the data presented is an *unstandardized* regression coefficient, what type of regression was used? Specify other: | | |  |
| TX_N | |  | | | 11) Treatment group sample size^[[8]](#endnote-8)^ | | |  |
| CON_N | |  | | | 11b) Control group sample size | | |  |
| TX_propN | |  | | | 12) Proportion of sample in treatment group (Tx/Tx+Control) | | |  |
| CON_propN | |  | | | 12b) Proportion of sample in control group (Con/Tx+Control) | | |  |
| TXMEAN | |  | | | 12) Treatment group mean (dependent variable) | | |  |
| CONMEAN | |  | | | 12b) Control group mean (dependent variable) | | |  |
| TXSD | |  | | | 13) Treatment group standard deviation (dependent variable) | | |  |
| CONSD | |  | | | 14) Control group standard deviation (dependent variable) | | |  |
| SUCCTX_N_a | |  | | | 15a) *n* of treatment group with successful outcome | | |  |
| SUCCCON_N_c | |  | | | 15b) *n* of control group with successful outcome | | |  |
| FAILTX_N_b | |  | | | 16a) *n* of treatment group with unsuccessful outcome | | |  |
| FAILTX_N_d | |  | | | 16b) *n* of control group with unsuccessful outcome | | |  |
| PROPTX_SUCCa | |  | | | 17) Proportion of treatment group with successful outcome | | |  |
| PROPCON_SUCCc | |  | | | 18) Proportion of control group with successful outcome | | |  |
| TVALUE | |  | | | 19) *t*-value (for independent/dependent-samples means comparisons only) | | |  |
| TVALUE_P | |  | | | 19b) *t-*test *p* value | | |  |
| ZVALUE | |  | | | 20) *z*-value | | |  |
| ZVALUE_P | |  | | | 20b) *z*-test *p* value | | |  |
| FVALUE | |  | | | 21) *F*-value | | |  |
| FVALUE_P | |  | | | 21b) *F*-test *p* value | | |  |
| CHISQ | |  | | | 22) Chi-square value (df=1) | | |  |
| CHISQ_P | |  | | | 22b) Chi-square *p* value | | |  |
| SD_X | |  | | | 23) Standard deviation of the independent variable | | |  |
| SD_Y | |  | | | 24) Standard Deviation of the dependent variable (note: for dichotomous dependent variables, this can be calculated using the formula √p(1-p)) | | |  |
| UNSTNDRGS | |  | | | 25) Unstandardized regression coefficient | | |  |
| STNDRGSS | |  | | | 26) Standardized regression coefficient | | |  |
| PRSONR | |  | | | 27) Pearson’s *r* | | |  |
| OTHDATA | |  | | | 28) Type of data effect size can be calculated from: (specify other—actual data) | | |  |
| **III. Conclusions made by the author** | | | | | | | |  |
| CNCLS_IMM | |  | | | 1) Did the assessment find evidence for the effectiveness of the treatment? (e.g., significant statistical test in the hypothesized direction)  0. No  1. Yes  2. Not tested | | |  |
| CNCLS_REL | |  | | | 2) Did the author(s) conclude there a relationship between the corporate crime prevention technique and a reduction in illegal corporate activities/violations, regardless of significant finding?  0. No  1. Yes  2. Can’t tell/Author did not discuss | | |  |
| CNCLS_ADD | |  | | | 3)Additional notes about conclusions: | | |  |
| UNIQUESAMPLE | |  | | | 4) Was this sample used in this study used in another article included in this meta-analysis? If yes, list other study IDs that use this sample. | | |  |
| CALC_ESd | |  | | | 29) Calculated effect size—mean difference | | |  |
| CALC_ESr | |  | | | 30) Calculated effect size—correlation | | |  |
| CALC_ODDS | |  | | | 31) Calculated effect size—Odds ratio | | |  |
| CALCES_LOGR | |  | | | 32) Calculated effect size (Logistic *r*) | | |  |
| CALCES_PROBD | |  | | | 33) Calculated effect size (Probit *d*) | | |  |
| CALCES_PROBR | |  | | | 34) Calculated effect size (Probit *r*) | | |  |
| CALC_ES_P | |  | | | 35) Calculated effect size—proportion(direct method) | | |  |
| CALC_ES_L | |  | | | 36) Calculated effect size—proportion(logit method) | | |  |
| CALC_UNBIAS_d | |  | | | 37) UNBIASED effect size—mean difference | | |  |
| CALC_UNBIAS_r | |  | | | 38) UNBIASED effect size—correlation | | |  |
| CALC_UNBIAS_OR | |  | | | 37) UNBIASED effect size—odds ratio | | |  |
| INVVRNCE | |  | | | 38) Calculated inverse variance weight | | |  |
| pooledSD | |  | | | 39) calculated pooled SD | | |  |
| STNDER | |  | | | 40) Calculated standard error | | |  |
| LowCI | |  | | | 41) Calculated lower bounds of Confidence Interval | | |  |
| hiCI | |  | | | 42) Calculated higher bounds of Confidence Interval | | |  |
| ES_notes | |  | | | 43) Notes on ES calculations | | |  |

1. **DECISION RULES AND NOTES ABOUT VARIABLES**

   If book or unclear, code from author bio [↑](#endnote-ref-1)
2. For our purposes, we will include studies that examine criminal and regulatory violations by corporations or their employees. The majority of corporate offenses are handled be regulatory agencies, like the EPA & OSHA. Thus, a focus on strictly criminal behaviors would limit this study and miss a great deal of corporate misconduct. According to Clinard and Yeager (1980), corporate crime is “any act committed by corporations that is punished by the state, regardless of whether it is punished under administrative, civil, or criminal law” (p. 16). **This offense-based definition encompasses a wide range of behaviors such as antitrust offenses, intentionally polluting the environment, unsafe labor practices, and tax and securities violations.**  [↑](#endnote-ref-2)
3. We are looking for variables that measure:

   Extralegal or legal interventions and that are policy-relevant (i.e., can be the subject of an intervention).

   “General organizational climate” is not relevant unless this includes specific policies in the organization that affect compliance.

   We are NOT interested in personality characteristics (e.g., morality) or a person’s approval of the law, job, policy, etc.

   Things we ARE interested in include

   Civil or criminal laws or sanctions (including civil cases)

   Ethical or safety **policies** within the company

   Internal compliance/monitoring programs

   Market devices such as shaming (e.g., bad publicity)

   Membership in external professional organizations that can sanction members

   Internal/external audits

   Corporate structure, including

   Insider vs. outsider members on the board of directors (including gray and independent directors)

   Public vs. private ownership

   Whether CEO is head of the board of directors

   People’s perceptions of risks (e.g., of getting caught or being sanctioned either formally or informally)

   We are mainly interested in the **presence vs. absence** of such variables, not descriptions about these IVs or gradations/dosage of the treatment (e.g., we are not interested in the size of the auditing company).

   If the independent variable is related to corporate compliance programs or something that seems to be of interest, include it only if you can dichotomize it and if there is not already a dichotomous variable of interest (e.g., company expenditures on compliance—could be dichotomized if companies report $0 versus non-zero values).

   When an intervention includes multiple components but only has one data point, just record one case and list all of the categories of the treatment variable under which it could fall, separate by commas. [↑](#endnote-ref-3)
4. Regarding measures of the dependent variable, we are not looking at overcompliance in and of itself.

   If overcompliance is measured, it can be used if compared to noncompliance (and should be combined with compliance if applicable).

   We ARE interested in severity measures (e.g., the amount of money lost, number of injuries) as well as compliance vs. noncompliance. [↑](#endnote-ref-4)
5. An illegal act is one that has been formalized as a law or regulatory statute—i.e., you can be sued, cited, or arrested for it.

   Unethical practices are those that are not punishable under the law but are morally questionable. [↑](#endnote-ref-5)
6. Decision rules on including ESs:

   If two or more tables/models are presented on the same IV and same operationalization of the DV, include all **unique** **measures** of the variables of interest.

   Prioritizing the table/model that 1) includes more IVs of interest and 2) has the full (more final) model.

   If alternative modeling strategies (e.g., OLS as well as Poisson) are used and there is no significant difference between the two use the simpler model.

   After including all of the variables of interest from the final model, include any other (not already included) variables of interest from other models that may have been dropped from the final model. [↑](#endnote-ref-6)
7. Anytime an article has more than one model, NOES_DTA should only have one value and there needs to be another case. **There needs to be a new case anytime you have a new independent variable, dependent variable, or model (e.g., anytime you have data coming from a different place).**

   - When both unstandardized and standardized coefficients, just record that you have standardized coefficients in NOES_DTA but record both in their appropriate places
   - When both means and t-tests, just record that you have a t-test in NOES_DTA but record both in their appropriate places
   - If you have a regression coefficient and descriptive statistics (means, SD), just record the regression in NOES_DTA but give all of the information in the appropriate place

   [↑](#endnote-ref-7)
8. For time-series, the baseline/pre-intervention numbers belong under the “control group.” The post-test is the treatment group. [↑](#endnote-ref-8)
